# Prediction of Associations between microRNAs and Gene Expression in Glioma Biology

**DOI:** 10.1371/journal.pone.0014681

**Published:** 2011-02-16

**Authors:** Stefan Wuchty, Dolores Arjona, Aiguo Li, Yuri Kotliarov, Jennifer Walling, Susie Ahn, Alice Zhang, Dragan Maric, Rachel Anolik, Jean Claude Zenklusen, Howard A. Fine

**Affiliations:** Neuro-Oncology Branch, National Cancer Institute, National Institutes of Neurological Disorder and Stroke, National Institutes of Health, Bethesda, Maryland, United States of America; University of Glasgow, United Kingdom

## Abstract

Despite progress in the determination of miR interactions, their regulatory role in cancer is only beginning to be unraveled. Utilizing gene expression data from 27 glioblastoma samples we found that the mere knowledge of physical interactions between specific mRNAs and miRs can be used to determine associated regulatory interactions, allowing us to identify 626 associated interactions, involving 128 miRs that putatively modulate the expression of 246 mRNAs. Experimentally determining the expression of miRs, we found an over-representation of over(under)-expressed miRs with various predicted mRNA target sequences. Such significantly associated miRs that putatively bind over-expressed genes strongly tend to have binding sites nearby the 3′UTR of the corresponding mRNAs, suggesting that the presence of the miRs near the translation stop site may be a factor in their regulatory ability. Our analysis predicted a significant association between miR-128 and the protein kinase WEE1, which we subsequently validated experimentally by showing that the over-expression of the naturally under-expressed miR-128 in glioma cells resulted in the inhibition of WEE1 in glioblastoma cells.

## Introduction

MicroRNAs (miRs) are small non-coding RNAs with mature transcripts of 18 to 25 nucleotides that have been implicated in the maintenance of the pluripotent cell state during early embryogenesis in mammals [1] as well as in tissue-specific or organ-specific development [2]. miRs interact with their target coding mRNA, inhibiting translation by degradation of the mRNAs, or blocking translation by direct and imperfect binding to the 3′ and 5′ un-translated regions (UTR) of targeted genes [3], [4], [5], [6], [7]. Furthermore, miRs exert control in combination with other regulatory elements such as transcription factors [8].

Focusing on cancer, over-expressed miRs might diminish the level of expression of targeted tumor suppressor genes - oncomirs - in tumors whereas miRs acting as tumor suppressors are silenced/down-regulated, leading to a higher expression rate of targeted oncogenes and contributing to the neoplastic process [9]. Additionally, miRs are frequently located in regions of loss of heterozygosity, genomic regions of amplification or common breakpoint regions [10] and have been identified to regulate the expression of tumor-associated genes in several tumors including glioblastomas (GBM) [9], [11], [12], [13].

Astrocytic tumors represent the most common form of glial tumors. According to the WHO classification [14], tumor anaplasia and aggressiveness increases from grades I to IV with glioblastomas (GBM-WHO grade IV) being the most malignant form of these tumors. Non-random genetic and epigenetic perturbations potentially lead to abnormal oncogene activation and/or tumor suppressor gene inactivation [14], [15], [16], [17], [18], [19].

Several studies have analyzed miR expression profiles in normal brain [1], [20], [21] and brain tumors [12], [13], [22], as well as tested their use as potential therapeutic tools [11], [22], [23]. Initial analysis of murine and human brain miRs predominantly indicated distinctive expression of miRs-9, -101, -124, -127, -128, -131 and -132 [1], [21]. Furthermore, alterations of miR-levels have been implicated in the de-regulation of critical players in major cellular pathways, modifying the differentiation, proliferation and survival of tumor cells. For instance, miR-7 and miR-221/222 were shown to be involved in the activation of the Akt and epidermal growth factor receptor (EGFR) signaling pathways [23], [24], [25], [26], [27] while miR-34a is a key downstream regulator of p53 [23], [24], [25], [26], [27].

miRs -10b and -21 have been consistently found highly over-expressed in astrocytic tumors [12], [13], [22] as well. Putatively, miRs-10b and -21 work as ‘oncomirs’ and decrease apoptosis in malignant cells. Down-regulated miRs-124 and -137 are involved in the differentiation of glioma stem cells [13], occurring with miR-128 that targets Bmi1 and E2F3a, promoting a pro-survival, undifferentiated self-renewing state [28], [29]. Regulation of both metabolic pathways in cancer cells and increase in their migration capabilities are also relevant properties that have been found to be controlled by miR-451 in glioma stem cell lines. Furthermore, miR-326 has been recently shown to regulate Notch-1 and -2 in such cells [30], [31].

Generally, miRs predominantly play an important role in signal transduction and regulation processes in various tumor types. To provide a better understanding of complex regulatory mechanisms that involve miRs, we computationally determined miRs that are significantly associated to expression changes of genes involved in signaling pathways of human gliomas. We combined data of physical interactions between miRs and the 3′UTR of mRNAs and gene expression profiles of 11 non-tumor control and 27 glioblastoma (GBM) samples. To assess the quality of our predictions we performed a high-resolution genomic analysis of the miR expression in the underlying tumor and control cases. Comparing in-silico predictions to our large-scale measurements we found that the combination of physical interactions of miRs and mRNAs and the expression change of the given genes indeed allowed an assessment of the influence of individual miR candidates on gene expression changes in the absence of any epigenetic effects and genomic alterations.

As an experimental proof of concept, we predicted associated miRs that influence the expression of WEE1, a tyrosine kinase that phosphorylates CDK1 [32], [33]. To validate our computational analysis and better understand the way WEE is regulated by miRs, we investigated the involvement of miR-128 and miR-27 and showed that expression of miR-128 is indeed strongly linked to the expression of mRNA and protein levels of WEE1.

## Results

### miR and mRNA expression in tumor samples

To investigate whether the expression levels of miRs have changed in glioblastomas we utilized 27 samples of glioblastoma (GBM) patients that belong to the GA subgroup [34] and 11 non-tumor control brain samples. 24 miRs had a lg_2_-fold-change FC of <−1, while we found 251 miRs with FC>1 (Table S1). As for the expression of mRNAs, we observed 1,495 over-expressed and 3,922 under-expressed mRNAs with |FC|>1 (Table S2).

### Computational prediction of associations between binding miR and expression of mRNAs

**Figure 1:**
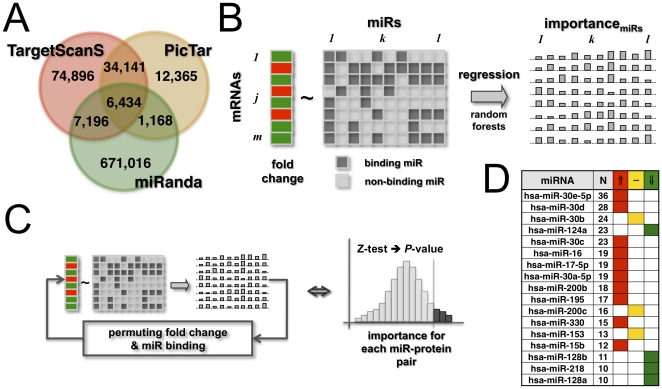
Determination of significantly associated miRs. In (**A**) we show the overlaps between sets of different sources of physical miR-mRNA interactions. Only accounting for potential interactions that at least were predicted by two methods we assembled 48,939 interactions. In (**B**) we assumed that the expression fold change of mRNAs is a function of the miRs that specifically bind the underlying mRNAs. As such, we represented the presence/absence of binding miRs as a binary (1/0) matrix, where each of the units is 1 if the corresponding miR can physically interact with the mRNA and 0 otherwise. mRNAs are represented as a vector of their corresponding expression fold changes. Utilizing random forest regression we determined miRs that are significantly associated with the fold change of mRNAs. Specifically, each pair of mRNA and miR is represented by a local importance measure reflecting the drop/gain of regression accuracy of a mRNAs fold change if the underlying miR is excluded. (**C**) Determining their statistical significance by a Z-test, we iteratively permuted the vector of fold changes and the binding matrix of miRs and calculated local importance values after each permutation step. (**D**) Utilizing a set of human genes that are involved human signaling pathways, we determined 128 miRs that are significantly associated to the fold change of mRNAs, comparing 27 GBM samples to non-tumor control cases (P<0.05). In the table we show miRs that have been found to be significantly associated to N≥10 mRNAs. We observed that such miRs are predominantly over-expressed(⇑, expression fold change FC>1), while a minority of miRs are under-expressed (⇓, FC<−1) or unchanged (∼, −1≤FC≤1) in GBMs.

Utilizing computational predictions from sources as algorithmically diverse as TargetScan [35], PicTar [36] and miRanda [37], [38], we assembled 48,939 interactions between 386 miRNAs and 6,725 mRNAs. In particular, we only selected interactions between miRs and mRNAs if they were at least predicted by two methods (Fig. 1A).

Our objective was to determine small sets of physically interacting miRs that are significantly associated to the expression of the underlying mRNA. As such, a significant association might indicate a significant role in the regulation of the underlying gene's expression. To identify groups of genes that were characterized by significant expression changes we assembled 184 annotated signaling pathways from the Pathway Interaction Database (PID) [39].

We calculated the mean fold change of all miRs that physically can interact with any given gene in the signaling pathways. Specifically, we found a weak inverse correlation (Pearson's r = −0.06, P<0.05), suggesting that putatively only a fraction of miR-mRNA interactions may play a role in the expression of the given genes. Identifying combinations of miRs that putatively are associated to altered mRNAs expression levels we applied the random forest algorithm [40], an ensemble-learning-algorithm that constructs regression trees with bootstrap data samples and random choices of predicting variables. We characterized each mRNA by its mean fold change and a x-dimensional binary vector (Fig. 1B). Referring to a miR, each of the x vector units is 1 if the corresponding miR can physically interact with the mRNA, and 0 otherwise. Applying the random forest algorithm, we performed a regression of the mean fold change of 1,277 mRNAs as a function of 355 interacting miRs. As a measure of a miRs impact on the regression process we assessed each miRs local importance for the fold change of each mRNA by randomizing mean fold change levels and interactions between miRNAs and mRNAs 100 times. Determining the significance of a miRs local importance with a Z-test (Fig. 1C) and correcting for multiple testing [41] we found 626 significant associations (P<0.05), involving 128 miRs that tuned the expression of 246 mRNAs (Table S3). Comparing our results, we utilized the HMDD database [42], that manually collects and curates associations of miRs and diseases from literature. In particular, we found a significant overlap of 8 miRs (P<10^−14^, hypergeometric test) out of 32 miRs that are associated with GBMs in HMDD.

In Fig. 1D, we show a subset of miRs that appeared most frequently in such associations (the full list is available in Table S4). Specifically, we counted the number of significant associations a given miR is involved in, allowing us to observe that such miRs are largely over-expressed in GBMs.

**Figure 2:**
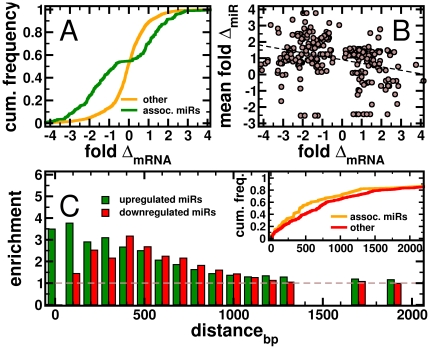
Characteristics of significantly associated miRs. (**A**) A sigmoidal curve described the fold change of all mRNAs that appeared in signaling pathways and were not bound by significantly associated miRs. Focusing on mRNAs that appeared to be regulated by significantly associated miRs, the corresponding distribution significantly shifted toward higher and lower fold changes. (**B**) For each mRNA, we calculated the mean fold change of all miRs that were significantly associated with a given mRNA. Specifically, we found that the expression fold change significantly decreased with increasing expression of miRs (Pearson's r = −0.30, P<10^−6^). In the inset of (**C**), we utilized the binding positions of miRs on the 3′UTR of a given mRNA and calculated the cumulative frequency of significantly associated miRs. In comparison to other miRs we observed an overrepresentation of associated miRs that bind near the start of the 3′UTR. Focusing on associated miRs, we determined the enrichment of over-expressed miRs (fold change>1) in sets of miRs that bind within a certain distance from the start of the 3′UTR. We clearly observe that over-expressed miRs tend to increasingly bind nearby the 3′UTR start. Considering under-expressed miRs (FC<−1), we find that the enrichment distribution peaks around 500 bp form the start of the 3′UTR.

In Fig. 2A, a sigmoidal curve described the fold change of all genes present in signaling pathways that were not significantly associated with miRs. Focusing on those mRNAs that are associated to miRs, we observed that the corresponding distribution strongly shifted toward lower and higher fold changes. In particular, the cumulative frequency distribution formed a plateau ranging from lg_2_-fold-changes −1 to +1, suggesting that associated miRs significantly changed the expression of the corresponding genes in GBMs. In a subsequent step, we calculated the mean fold change of all miRs that were significantly associated with the underlying mRNAs. In Fig. 2B, we found that the expression fold change of mRNAs is significantly correlated with the expression of its associated miRs (Pearson's r = −0.30, P<10^−6^), a remarkable 5-fold increase, demonstrating our ability to identify miRs that potentially play a role in the expression of given genes.

To better understand where significantly associated miRs actually bind mRNAs we calculated the cumulative frequency of significantly associated miRs as a function of their distance to the start of the 3′UTR. Considering sequence alignments of a given 3′UTR and a miR, we defined the position of the first aligned nucleotide of the 3′-UTR as the distance to the start of the un-translated region. In the absence of splice-version specific sequence data of 3′UTRs in GBMs we accounted for all alignments of miRs and un-translated regions of a given mRNA. In comparison to non-associated miRs we observed an enrichment of associated miRs that bind near the start of the 3′UTR of the corresponding mRNAs (inset, Fig. 2C). Assuming that the placement of binding sites of significantly associated miRs was a non-random process we hypothesized that such binding sites might increasingly be occupied by over(under)-expressed miRs. Therefore, we determined the enrichment of over-expressed miRs (fold-change FC>1) in sets of all miRs that interacted within a certain distance from the start of the 3′UTR. In Fig. 2C, we observed a strong tendency of over-expressed miRs to increasingly bind nearby the 3′UTR start. In addition, we observed a similar result for under-expressed miRs, (FC<−1). However, the distribution is shifted several 100 bp away from the start of the 3′UTR. Assuming that their efficacy was mediated by occupying binding sites nearby the stop codon, miRs might spatially block ribosomes from finishing translation and therefore avoid degradation of the underlying mRNA [43]. Our results suggest that over-expressed miRs, corresponding to under-expressed genes, potentially utilize these proximal positions on the 3′-UTR, thereby allowing under-representation of those mRNAs.

**Figure 3:**
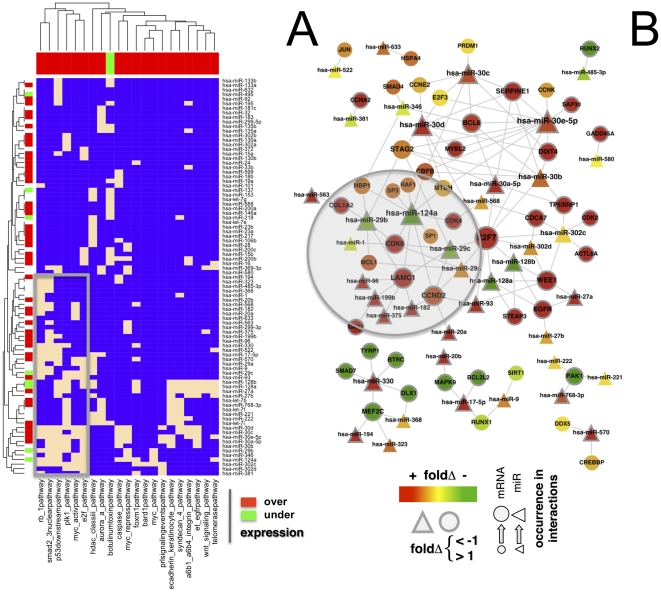
Significantly associated miRs in signaling pathways. (**A**) Utilizing all significant associations between 128 miRs and 246 genes, we constructed a bipartite matrix between miRs and signaling pathways that are over(under)-expressed in human GBMs. We established a link if the sets of genes in a pathway overlapped with mRNAs that are associated with a certain miR. Specifically, we found 21 pathways that are largely over-expressed in GBMs and 87 miRs. While a majority of miRs ware over-expressed (expression fold change FC>1), we found a small minority of miRs that was under-expressed in GBMs (FC<−1). In particular, we highlighted a small cluster that pooled most of the under-expressed miRs and prominent pathways such as the p53 downstream and myc activation pathway (box). In (**B**) we mapped all interactions between associated miRs and genes that appeared in the corresponding pathways. Confirming our predictions, we found significant interactions between genes of the extracellular matrix and miR-29bc and -124a that have been previously implicated in glioblastomas and other cancer types (shaded area). Furthermore, miR-124a was previously reported as a regulator of CDK6 in GBMs.

Utilizing all 626 significant associations between 128 miRs and 246 genes, we constructed a bipartite matrix between miRs and signaling pathways if a given pathway shared at least one gene with the associated targets of a miR. Narrowing our focus, we considered signaling pathways that are over(under)-expressed in GBMs. In particular, we applied GSEA [44], allowing us to find 21 enriched signaling pathways (P<0.05) that are largely overrepresented in GBMs. Among such enriched pathways we found several prominent signaling pathways that have been implicated in tumor biology. We established a link if pathways shared at least one gene with associated targets of a miR. While a majority of 87 miRs thus obtained were over-expressed (expression fold change FC>1), we found a small minority of miRs that were under-expressed in GBMs (FC<−1). Hierarchically clustering the bipartite matrix, we observed two large clusters of pathways as well as 2 large clusters of miRs. Specifically, we highlighted a dense cluster that pooled most of the under-expressed miRs and prominent pathways such as the p53 downstream and myc activation pathway (Fig. 3A). Mapping all associations of miRs and genes that appeared in the corresponding pathways, we found significant interactions of prominent miRs (Fig. 3B). Specifically, miR-124a was previously reported as a regulator of CDK6 in GBM [13] and medulloblastoma [45]. Furthermore, we predicted that both miR-29b and -29c were strongly associated with extracellular matrix proteins such as LAMC1 and COL1A2. Previous reports confirmed that these miRs regulate the expression of extracellular matrix proteins in nasopharyngeal carcinomas [46], contributing to positive regulation of osteoblast differentiation [47] and playing an important role in cardiac fibrosis [48].

### WEE1 is over-expressed in GBM and TIC

**Figure 4:**
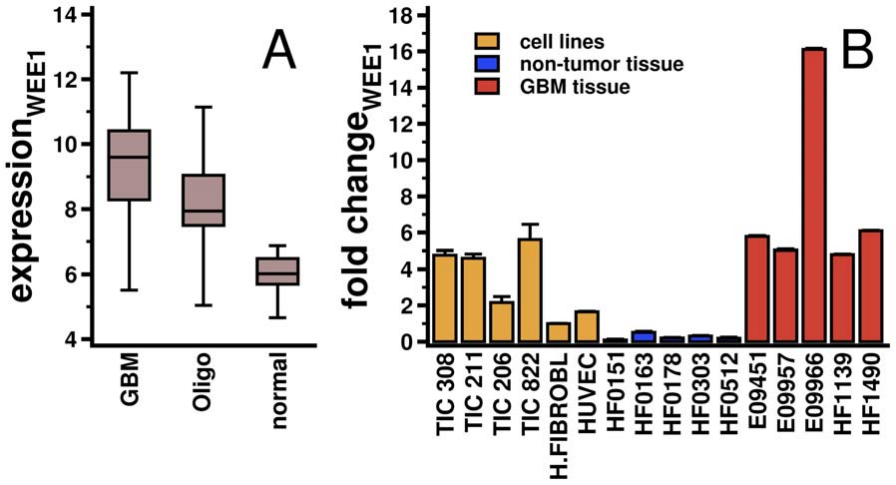
Expression patterns of WEE1. (**A** WEE1 is over-expressed in GBMs and Oligodendrogliomas. (**B**) Expression of WEE1 was validated by quantitative reverse transcription polymerase reaction (RT-qPCR) in five representative GBM tumor samples that belong to the GA subtype, five non-tumor samples, four tumor initiating/stem cell lines (TICs) and two unperturbed cell lines (human fibroblasts and HUVEC). We observed that TIC 308 showed an average WEE1 fold change that was similar to the corresponding averages in the GBM samples.

As an experimental proof of concept, we predicted significantly associated miR interactions that influence the expression of WEE1, a tyrosine kinase that phosphorylates CDK1 at the tyrosine-15 (CDK1-Y15) position [32]. Previously published data suggested that over-expression of WEE1 is critical for the viability of some cancer types, and cell lines displaying higher levels of WEE1 expression are sensitive to WEE1 inhibition [33]. Utilizing expression data from primary glial tumors, we confirmed that WEE1 is strongly over-expressed in gliomas compared to non-tumor control cases (Fig. 4A). To validate our computational analysis and better understand how WEE1 is regulated by miRs, we first assayed the absolute levels of WEE1 mRNA expression in 5 GA subgroup GBMs, 5 non-tumor brain samples, four GBM-derived tumor initiating/stem cell lines (TICs) and two normal cell lines (human fibroblasts and HUVEC), using quantitative reverse transcription polymerase assays (RT-qPCR). As seen in Fig. 4B, the RT-qPCR data demonstrated that WEE1 mRNA is over-expressed in both GBMs and TIC lines compared to non-tumor controls. Since the WEE1 genomic locus is not placed in an area of chromosomal number alteration (CNA) in any of our glioma specimens we ruled out that the levels of WEE1 gene expression are simply explained by alterations of gene copy numbers (data not shown).

**Figure 5:**
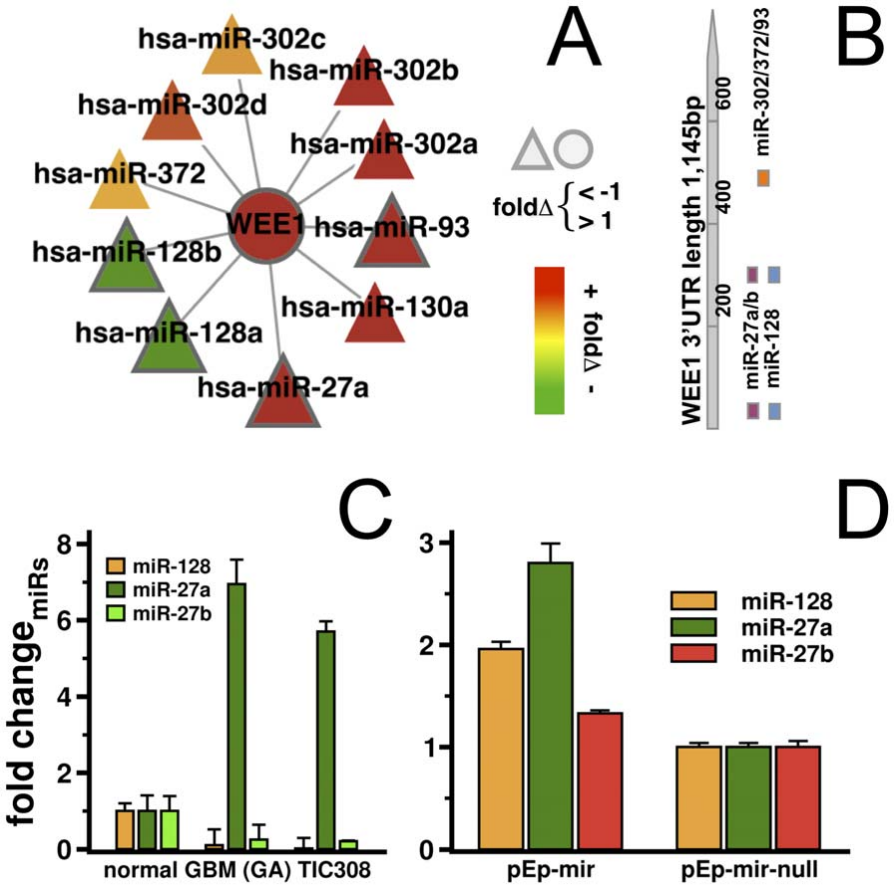
Significantly associated miRs of WEE1. In (**A**) we show all significantly associated interactions between the mRNA of WEE1 and miRs where miR-128ab were under-expressed (fold change FC<−1) and miR-27a/93 were over-expressed (FC>1) in our GBM samples. (**B**). Corresponding miR binding sites in the WEE1 3′UTR are located in three main binding areas within the first 500bp from the 3′UTR start. Specifically, miR-128/27 have two binding sequences around nucleotides 15 and 236 while miR-302abcd/372/93 potentially recognize a common binding site around nucleotide 465. (**C**) Using RTqPCR TaqMan assays, we detected that miR-128/27b were under-expressed and miR-27b was strongly over-expressed in tumor samples. We found a similar miR expression profile in the tumor initiating/stem cell line, TIC308, where miR-128 and miR-27b kept their low expression levels. (**D**) Transfection of miR-specific expression vectors of TIC308 cells allowed the recovery of miR-128/27b levels as measured by RTqPCR.

We found 36 miRs that were predicted to interact with WEE1 mRNA based purely on their seed sequence in the WEE1 3′ UTR. However, our analyses demonstrated just 10 significantly associated miR/WEE1 mRNA interactions in our GBM samples (Fig. 5A). Among our set of associated miRs, we discovered an accumulation of binding-site sequences within the first 500 bp of the WEE1 3′UTR region (Fig. 5B). miR-128 and miR-27ab were among these miRs that bind nearest to the start of the 3′UTR, an interesting observation given that miR-128 has been shown to target Bmi1 and E2F3a, thereby promoting an undifferentiated self renewal state in glioma cells [28], [29]. Utilizing RTqPCR TaqMan assays we validated that miR-128 and miR-27b were highly under-expressed in our GBM samples and 308 TIC cell line whereas miR-27a was over-expressed (Fig. 5C).

### WEE1 is a direct target of miR-128 and miR-27 and affects cell cycle progression

**Figure 6:**
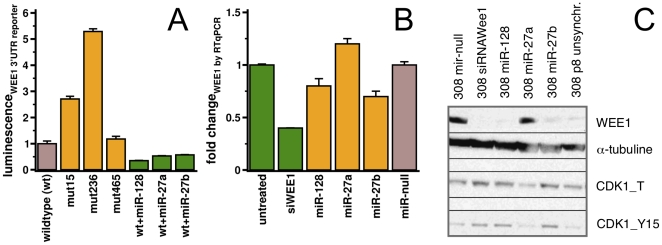
Effects of significantly associated miRs on WEE1 expression. (**A**) miRs-128/27a/27b that are under-expressed in glioma were transiently over-expressed in TIC308 cells with corresponding pEp-miR-expression vectors. Co-transfecting this cells with a luciferase reporter constructs that contained the whole UTR region of WEE1, we observed decreasing luciferase activity (brown/green bars). Furthermore, co-transfection with reporters containing mutated sequences of seed regions in the three potential binding sites around nucleotides 15, 236 and 465 of the WEE1 3′UTR (yellow bars) showed an increase in luciferase activity when binding sites 15 and 136 of miR-128/27 were mutated. However, we did not find an effect when position 465 was mutated where binding of the naturally over-expressed miRs-302/372/93 was predicted. Our results allowed us to conclude that miRs-128/27a/27b indeed bind the 3′UTR of WEE1, and their low expression in gliomas potentially plays a crucial role in the high levels of WEE1 expression in these tumors. (**B**) Similarly to silencing WEE1 with a corresponding siRNA, ectopic expression of miR-128 and miR-27b reduced WEE1 mRNA and protein levels in synchronized cells of TIC308. (**C**) Downstream effects of WEE1 modulation by miRs was observed as CDK1_Y15 phosphorylation is similar compared to when WEE1 was directly down-regulated by a specific siRNA treatment. In particular, we observed that the recovery of miR-128 as well as over-expression of miR-27b constrained WEE1 expression.

We transiently transfected the naturally under-expressed miR-128 and miR-27a/b along with a WEE1 3′-UTR luciferase reporter construct into TIC308 cells to experimentally verify their binding to the WEE1 3′UTR (Fig. 5D). Expression of all three miRs significantly downregulated the luciferase activity (Fig. 6A). In contrast, mutations of the miR-128 and 27a/b binding nucleotides 14 and 236 of the 3′-UTR relieved the miR-mediated repression of luciferase activity. This effect even held when these miRs were transiently over-expressed, indicating the specificity of the miR impact.

On the other hand, mutations of the binding site around nucleotide 465 have the lowest relieving effect, confirming that the highly over-expressed miR-93 that binds this downstream site has a lower effect on expression. Over-expression of miR-128 and miR-27b directly reduced WEE1 mRNA and protein levels in synchronized cells of TIC208 (Fig. 6BC). Consistent with a significant biological effect of miR-128 and miR-27b knockdown of WEE1, we observed a corresponding increase in CDK1-Y15 phosphorylation similar to the effects, following down-regulation of WEE1 by a specific siRNA treatment (Fig. 6C).

## Discussion

Although a growing appreciation of the importance of miRs in cancer biology is emerging, much remains to be learned about their roles in specific regulatory programs. Utilizing GBM samples, we showed that the mere knowledge of physical interactions of miRs and the expression change of the underlying interacting mRNAs allows a prediction of associated miRs that drive the expression of their targets. Utilizing random forests, an ensemble machine learning approach, we determined combinations of significantly associated miRs that contributed to the expression fold change of the underlying targets. At this point of the analysis, we deliberately refrained from using large expression data sets of miRs and focused entirely on physical interactions between un-translated regions of mRNAs and miRs. Subsequently, we utilized large-scale expression data of miRs to assess the quality of our results, allowing us to observe that under- and over-expressed miRs are predominantly interacting with over- and under-expressed genes. Furthermore, we observed that significantly associated interactions were characterized by an inverse relationship between expression levels of a specific miR and its target mRNA. This result is consistent with existing models, suggesting that mRNAs are usually under-expressed following binding of certain miRs. Accordingly, our associated interactions allowed us to find that such over-(under)expressed miRs predominantly interacted through binding sites that were placed near the start of the 3′UTR of the target mRNA.

In an attempt to validate one such computationally derived association, we utilized WEE1 as a representative example and found 10 miR candidates out of a pool of 36 miRs. Generating a small tractable set of testable hypotheses our computational analysis constrained the pool of regulatory candidates by more than 60%. Experimentally, we demonstrated that WEE1 is predominantly influenced by miR-128/27b in a sequence-specific manner following binding to sites nearby the start of the WEE1 3′UTR. Consistent with our results, miR-128 has been previously described as down-regulated in gliomas [12], playing a potential role in tumor biology by targeting the transcription factors E2F3a [28] as well as Bmi-1 [29]. Similarly, a critical effect on cell division mediated by WEE1 was observed in hESC [49] where miR-195 expression in Dicer-knockdown cells rescued cell cycle kinetics by directly targeting WEE1 3′UTR. Finally, the necessity for WEE1 in cell division has recently been described in primary fibroblasts [50], and its specific importance in human glioblastoma has been demonstrated thereby independently validating our computational findings [51].

Additionally, miR-124 has also been extensively implicated in glioma pathogenesis [13], [23] although the association with extracellular matrix proteins expression presented in our study is completely novel, necessitating further exploration of their biological relevance.

In summary, we have shown that the mere knowledge of physical interactions between specific mRNAs and miRs can be used to predict putative causal regulatory interactions in human tumor specimens. However, we have to stress that we assessed the influence of individual miR candidates on expression changes of genes assuming the absence of any epigenetic effects and genomic alterations. In this light, we have found evidence suggesting that miRs that interacted more proximally in the 3′UTR, near the translation stop site, may have greater regulatory effects on mRNA levels than those that bind more distally. Finally, our analysis allowed us to predict and subsequently validate an association between miR-128/27b and the protein kinase WEE1, a protein of central importance in cellular proliferation and survival, demonstrating the potential power of this computational approach.

## Materials and Methods

### Tumor and Tumor Initiating Cell (TIC) samples

After written consent tumor samples were obtained from patients undergoing surgery at the National Institutes of Health (NIH) in accordance with the surgical procedures of the National Cancer Institute's Institutional Review Board that specifically approved this study. We used 27 samples that were provided as snap frozen sections. Utilizing a computational classification scheme [34], we confirmed that these samples were members of the GA subgroup of glioblastomas. As a control, 11 non-tumor samples (temporal lobe resection of epileptic patients) were analyzed concurrently to provide a baseline for the miR/mRNA expression values. Procedures regarding the derivation of TICs were described previously [52].

### Total RNA extraction

Following the manufacturer's instructions, 100 mg of tissue were used to extract total RNA using the Trizol Plus isolation protocol (Invitrogen, Carlsbad, CA). While RNA quantity was determined using the NanoDrop® ND-1000 spectrophotometer the integrity of the RNA was verified with the Bioanalyzer System (Agilent Technologies, Palo Alto, CA) using the RNA Pico Chips with a RIN>7.

### miR profiling and statistical analysis

miR expression in 27 glioblastoma tumors and 11 non-tumor brain cells was profiled using the NCode™ Multi-Species miRNA Microarray v2 (Invitrogen Corp.) which contains ∼1,100 unique probes printed in triplicates for detecting validated miRs in *H.sapiens, M. musculus*, *R. norvegicus*, *C. elegans*, *D. melanogaster* and Zebrafish. 553 probes were designed to detect human miRs (ver. Sanger 9.0).

Extracted RNA was labeled with Alexa Fluor® Dye using NCode™ miR Labeling System and hybridized to species-specific antisense miR probes on the array. Quality of all arrays was initially checked by the internal Alexa fluor dye control. Arrays were analyzed using GenePix Pro 6.0, and .gpr files for individual arrays were generated. After correcting background noise by subtracting median background signals, data were normalized using Loess method in Partek Genome Suite version 6.5.

### mRNA profiling and data treatment

Utilizing T7-linked Oligo (dT) primer we converted 6 µg of total RNA to cDNA with superscript reverse transcriptase (Invitrogen) and *in-vitro* transcribed complementary DNA with the T7 Bioarray High Yield RNA Transcript Labeling Kit (ENZO Diagnostics) to generate biotinylated cRNA. 20 µg of purified cRNA were fragmented and hybridized to the Genechip® Human Genome U133 Plus 2.0 Expression arrays (Affymetrix, Inc., Santa Clara, CA). Following the manufacturer's recommendations the arrays were processed using fluidics station 450 and high-resolution microarray scanner 3000. Finally, initial gene expression analysis data files were generated using Affymetrix GeneChip Operating Software (GCOS) version 1.3.

Utilizing parameters in .rpt files generated by GCOS, all arrays were checked if they complied with minimal quality control standards. Specifically, we tested if a scaling factor is <5 when the expression values are scaled to a target mean signal intensity of 500. Similarly, we controlled that the signal intensity ratios of the 3′ to 5′ end of the internal control genes of ß-actin and GAPDH is <3. As a final requirement, Affymetrix spike controls (BioC, BioDN and CreX) were always present with present call rates of >35 % for brain tissue. Arrays that passed the minimal quality control were normalized at the PM and MM probe level using dChip [53]. Using the average difference model to compute expression values, model-based expression levels were calculated with normalized probe level data, and negative average differences (MM>PM) were set to 0 after log-transforming expression values.

While the normal specimen section came from non-tumor bearing patients, we demanded that the behavior of the global gene expression profiles had to resemble the normal tissue in exploratory data analysis of microarrays using principle component analysis (PCA) and hierarchical clustering (HC). Accounting for weak signal intensities, all probe sets with more than 10% of zero log-transformed expression values were removed. Representing each gene, we chose the corresponding probe set with the highest mean intensity in the tumor and control samples.

### miR-mRNA Interactions

Available prediction methods have strongly varying degrees of sensitivity and specificity. Therefore, we assumed that a combination of methods profoundly mitigates the problem of picking up false positives and negatives and only accounted for potential interactions that at least were predicted by two algorithmically different methods. We assembled 48,939 interactions between 386 miRNAs and 6,725 mRNAs, utilizing human specific data from PicTar [36], miRanda [37], [38] and TargetScanS [35].

### Molecular Pathways

As a comprehensive collection of human signaling pathways we utilized pathway information from the NCI/NIH/Nature Pathway Interaction Database (PID) [39]. Specifically, PID provides information about 184 different human signaling pathways.

### Random Forests

Random Forests is an ensemble learning method [40] where regression trees are constructed using N different bootstrap samples of the data (‘bagging’). In addition, random forests change how regression trees are constructed by splitting each node, using the best among a subset of M predictors randomly chosen at that node (‘boosting’), and new data is predicted by aggregating the predictions of N trees.

Fitting the fold change of mRNAs in GBM samples as a function of miRs that bind the 3′UTR of the underlying mRNAs, we represented each mRNA by a fold change value und a variable list that contains 1's, if the corresponding miR can bind the underlying mRNA and 0 otherwise. Growing 50,000 trees, we sampled 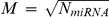 out of all N miRs (i.e. variables).

The importance of a miR for the regression process can be assessed by the increase of the prediction error when out-of-bag data (i.e. data not in the bootstrap sample) is permuted. Specifically, we utilized the local importance that reflects the influence of a miR on the fold change of the underlying mRNA. Assuming that only a subset of miRs significantly contributed to the fit, we assessed each miRs local importance by a permutation analysis. Randomizing binding miRs and expression fold changes of mRNAs 100 times, we determined the average importance 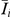 and standard deviation 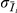 of each miR *i*. Subsequently calculating a miRs score by 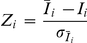, we determined it's statistical significance by a one-tailed p-value from a standard normal cumulative distribution function. We corrected P-values using [41] and collected all interactions between miRNAs and mRNAs with P<0.05.

### Enrichment

To obtain an estimate if over(under)-expressed miRs (fold change |FC|>1) predominantly bind nearby the start of the 3′-UTR, we calculated the corresponding fraction of such miRs that bind within a distance *d* as 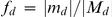, where *M_d_* is the number of all miRs within distance *d* from the start of the 3′-UTR in the underlying sample. As a null hypothesis, we randomly picked sets of over(under)-expressed genes represented by the fraction 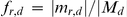. We defined the enrichment of over(under)-expressed genes that bind within a distance *d* as 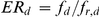 and found an enrichment if *ER_d_>1* and *vice versa*.

### Transfections

TIC308 cells were transfected using the mouse NSC Nucleofector kit (Amaxa, Cologne, Germany) program A-33. miR expression vectors for the induction of miRs-128/27a/27b (pEp-hsa-mir-vectors, Cell Biolabs, San Diego, CA) or miR-null negative controls were used at 2 µg per transfection. For small interfering RNA (siRNA)-mediated target knockdown of WEE1, four siRNAs in the ON-TARGETplus SMARTpool L-005050-00-0005 were used (Thermo Fischer Scientific, Lafayette, CO) with a final concentration of 3 pmol of each duplex. Transfection efficiency was measured using GFP max vector (Amaxa, Cologne, Germany) in every condition.

### Luciferase assay

miR expression vectors for the induction of miR-128/27a/27b (Cell Biolabs, San Diego, CA) were co-transfected with WEE1 3′ UTR/ Empty UTR Luciferase reporter vector (SwitchGear Genomics, Merlo Park, CA) using mouse NSC Nucleofector kit (Amaxa, Cologne, Germany) in TIC308. Also, seed regions in the WEE1 3′UTR were mutated and cloned into WEE1 3′ UTR Luciferase reporter vector (SwitchGear Genomics, Merlo Park, CA) using the QuikChange II XL mutagenesis kit (Stratagene). Specifically, we generated mutants for binding sites close to nucleotide 15 (CCTGAACACTGTGA to CCTGAACgggcTGA), nucleotide 236 (GGTTAACCACTGTG to GGTTAACCgggGTG) and nucleotide 465 (TGGGAGCACTTTG to TGGGAGCACgggG).

72 hours post-transfection GFP+ cells were sorted by fluorescence-activated cell-sorting (FACS) and/or incubated with Hoechst 33342 and propidium iodide for cell cycle analysis. Luciferase expression was measured in GFP+ cells using the Steady-Glo Luminescence kit (Promega, Madison, WI) after 48 hours.

### mRNA/miR and protein levels

Expression of WEE1 and GAPDH genes as well as miRs of interest were analyzed using specific TaqMan® Assays in the 7900HT Real Time PCR system (Applied Biosystems, Foster City, CA) following standard protocol. Synchronized cells were collected for protein lysates 72 hours post-transfection. Protein levels were measured with antibodies against WEE1 (sc-5285), CDK1-total (Cell Signaling, Beverly, MA, #9112) and Phospho-CDK1 (tyr15) (Cell Signaling, Beverly, MA #4569). An anti-tubuline antibody was used to test equal protein loading.

## Supporting Information

Table S1 List of 462 miRs and their lg_2_-fold change of expression in 27 GBM and 11 non-tumor samples.
(0.05 MB XLS)

Table S2 List of 20,288 genes and their lg_2_-fold change of expression in 27 GBM and 11 non-tumor samples.
(1.31 MB XLS)

Table S3 List of 626 predicted associations between mRNA and miR (P<0.05). We indicated if the underlying miRs and mRNAs are over (⇑, lg_2_ fold change>1) or under (⇓, lg_2_ fold change<−1) expressed in GBMs.
(0.07 MB XLS)

Table S4 List of 128 miRNAs that significantly interact with a gene (P<0.05). In particular, we counted the number of significant interactions (N) and indicate if the underlying miR is over (⇑, lg_2_ fold change>1) or under (⇓, lg_2_ fold change<−1) expressed or largely unchanged (∼) in GBMs.
(0.03 MB XLS)
